# Primary small-cell neuroendocrine carcinoma of the duodenum – a case report and review of literature

**DOI:** 10.1186/1477-7819-2-28

**Published:** 2004-08-15

**Authors:** Naohiro Sata, Munetoshi Tsukahara, Masaru Koizumi, Koji Yoshizawa, Katsumi Kurihara, Hideo Nagai, Tsutomu Someya, Ken Saito

**Affiliations:** 1 Department of Surgery, Jichi Medical School, 3311-1 Yakushiji Minami-kawachi Tochigi, Japan; 2 Department of Pathology, Jichi Medical School, 3311-1 Yakushiji Minami-kawachi Tochigi, Japan

## Abstract

**Background:**

Small-cell neuroendocrine carcinoma in the duodenum is an extremely rare neoplasm with poor prognosis.

**Case presentation:**

A 57-year-old man presented with sudden onset gastrointestinal bleeding and fainting attacks. Duodenoscopy and hypotonic duodenography revealed a 3 × 3 cm protruding tumor with ulcerations situated opposite the ampulla of Vater in the second part of the duodenum. Local excision of the tumor was performed, followed by adjuvant chemotherapy with 5-fluoro uracil and leucovorin. Examination of the tumor by immunohistochemistry and electron microscopy indicated it to be neuroendocrine in nature, expressing synaptophysin and AE1/AE3, and containing dense core granules. The patient showed no sign of recurrence and has been disease-free for more than 48 months after surgery.

**Conclusions:**

Most cases of small-cell neuroendocrine carcinoma in the duodenum show rapid progression of the disease, and even radical surgery with or without chemotherapy do not prevent death. We report a rare subtype of small-cell neuroendocrine carcinoma. This subtype appears to have a much better prognosis, and may be amenable to local excision, if the lesion is away from the ampulla of Vater.

## Background

Duodenal Neuroendocrine tumors constitute 5% of all gastrointestinal neuroendocrine tumors [1,2]. Most of these show well-differentiated features and are classified as carcinoids or somatostatinomas [3-6]. Occurrence of carcinoma is rare, and carcinomas with anaplastic character, which are classified as small-cell carcinomas, are even less frequent [7-12]. The most common small-cell neuroendocrine carcinoma (NEC) is the small-cell undifferentiated carcinoma of the lung [13,14]. Although the features of these pulmonary tumors are well defined, the characteristics of their extrapulmonary counterparts are still unknown. We report a case of small-cell NEC in the duodenum that had unique morphological features and exceptionally good clinical outcome.

## Case presentation

**Figure 1:**
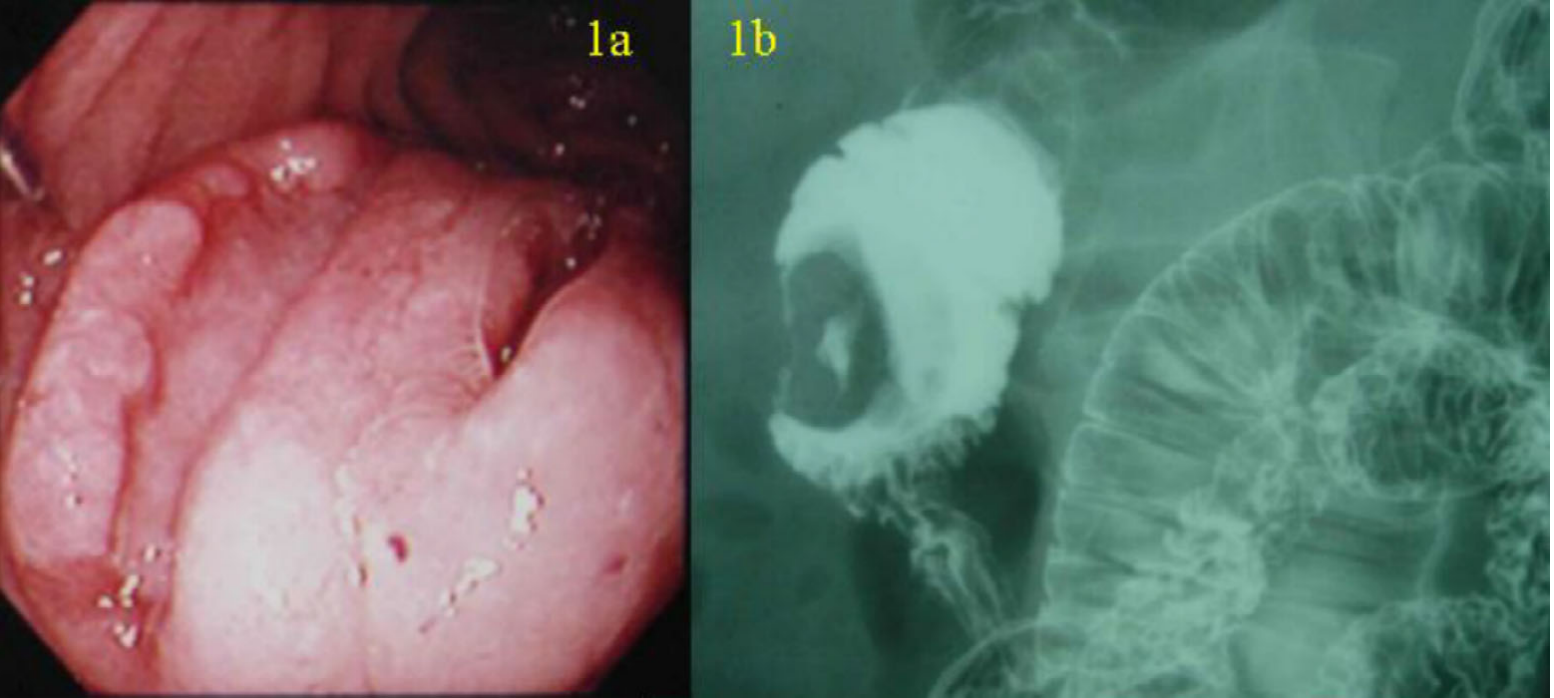
(a) Duodenoscopy showing a 3 × 3 cm protruding tumor with two ulcerations located opposite the ampulla of Vater in the second portion of the duodenum. (b) Hypotonic duodenography showing the donuts-shape tumor in the duodenum.

A 57-year-old man presented with sudden gastrointestinal tract bleeding and episode of fainting. Duodenoscopy (Figure 1a) and hypotonic duodenography (Figure 1b) revealed a 3 × 3 cm protruding tumor with two ulcerations located opposite the ampulla of Vater in the second part of the duodenum. Laboratory data showed no abnormalities in blood chemistry, tumor markers (CEA, CA19-9, NSE, proGRP) and endocrine markers (somatostatin, gastrin, glucagons, serotonin, VIP) except a moderate anemia (9.5 g/dl hemoglobin). No abnormal findings were observed in the chest X-ray and computed tomography (CT).

**Figure 2:**
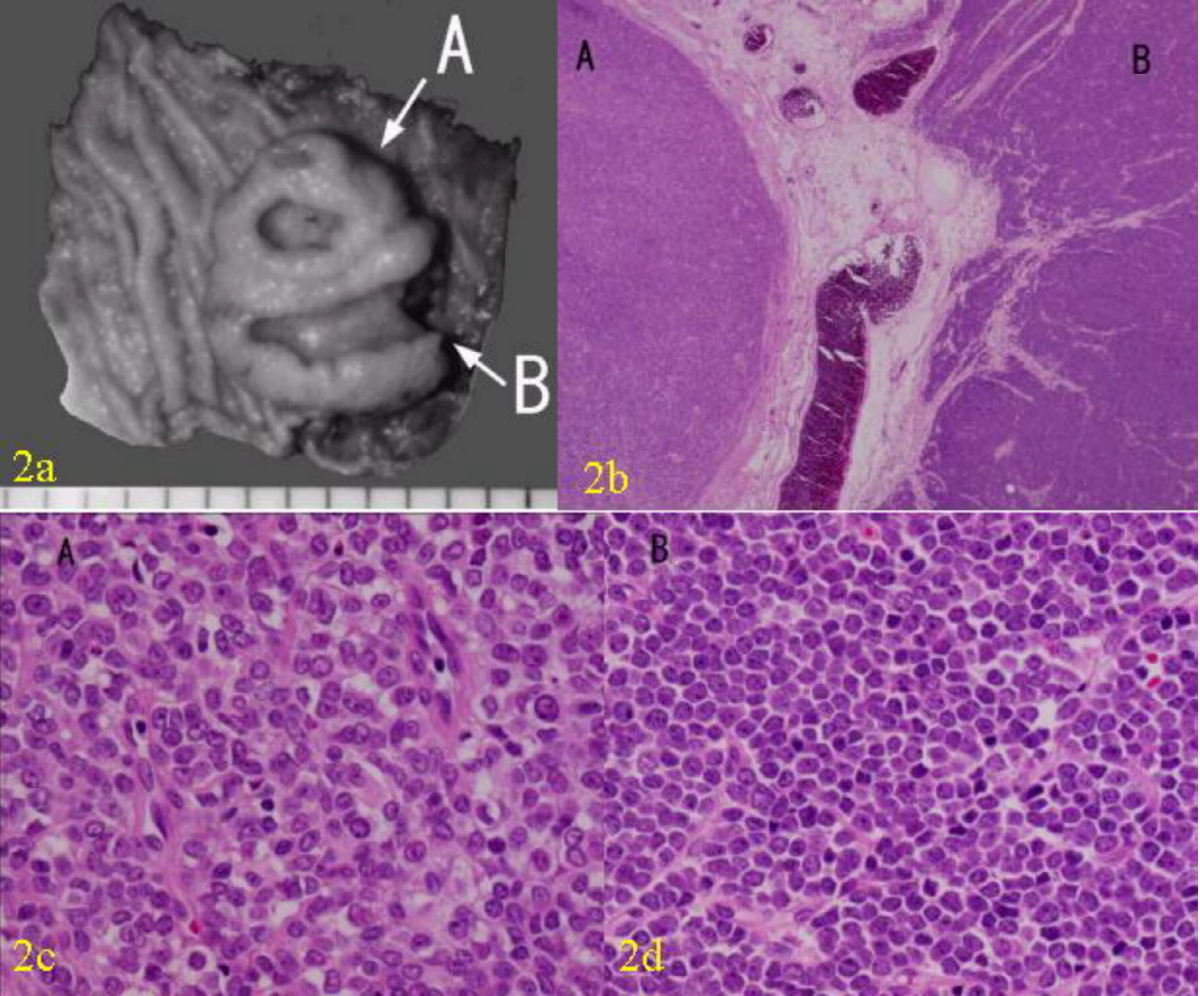
Macroscopic and microscopic findings of the tumor. (a) Gross appearance of the tumor. The tumor was divided into two components, component A (round shape) and B (crescent shape). (b) Photomicrograph of the gross appearance of the tumor (Hematoxylin and eosin X 2). (c) Photomicrograph of the component A showing fibrous tissue, small nuclei, and clear nucleoli. (Hematoxylin and Eosin X 40). (d) Photomicrograph of the component B showing more anaplastic features typical of small-cell carcinoma, such as sheets of tightly packed anaplastic cells with round nuclei and scanty cytoplasm. (Hematoxylin and Eosin X 40).

**Figure 3:**
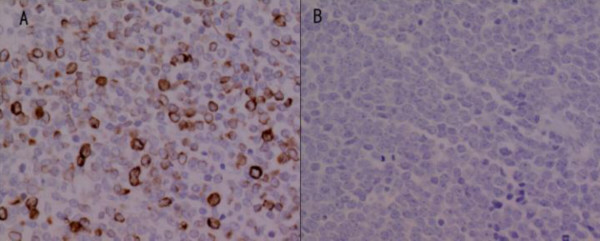
Immunostaining for AE1/AE3 showing (a) diffuse cytoplasmic positivity in the component A, and (b) no reactivity in the component B.

**Figure 4:**
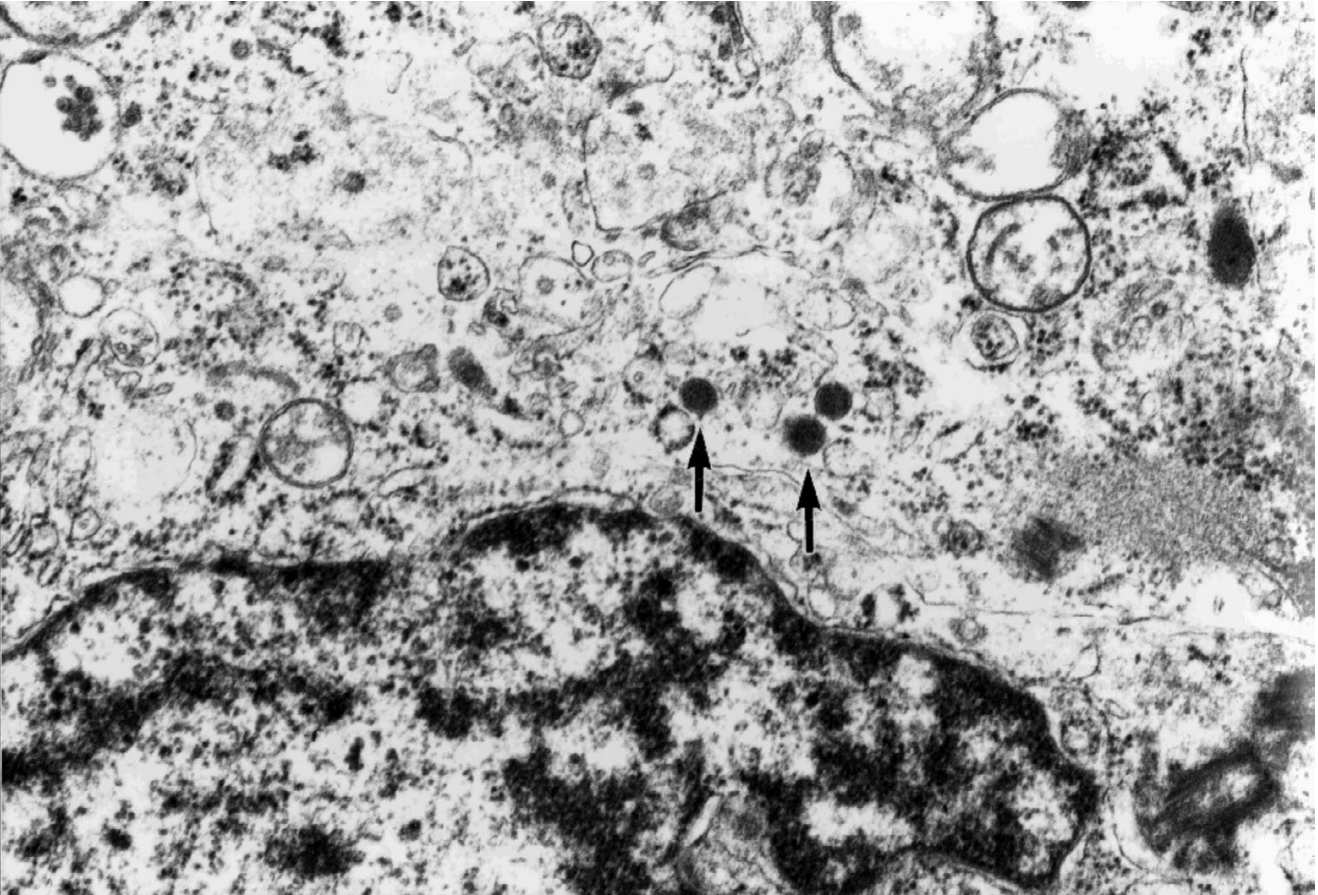
Ultrastructural study showed cytoplasmic dense-core granules in the component A.

A laparotomy was performed. As there was no serosal invasion or regional lymphadenopathy wide local excision of the tumor was performed. On gross examination, the tumor showed two ulcerations and two different morphological components (Figure 2a and 2b). One component (component A) was round in shape with a round ulceration on the top, and the other component (component B), which enclosed the round component, was crescent in shape with a spindle-shaped ulceration on the top. The two components showed different histopathological and immunohistochemical features (Table 1). The round component contained fibrous tissue, small nuclei, and clear nucleoli. Histopathologically, the crescent component had more anaplastic features typical of small-cell carcinoma, such as sheets of tightly packed anaplastic cells with round nuclei and scanty cytoplasm (Figure 2c, 2d). Neuroendocrine differentiation was investigated using immunohistochemical and ultrastructural techniques. Both components showed neuroendocrine features, with immunochemistry identifying synaptophysin and AE1/AE3 (Figure 3a and 3b), and electron microscopy identifying dense core granules (Figure 4). Immunochemistry also showed that the crescent component expressed less cytokeratin, vimentin and CD56, and more MIB-1 than the round component.

**Table 1:** Immunochemical characteristics of the two components of the tumor.

		**Synaptophysin**	**AE1/AE3**	**Vimentin**	**CD56**	**chromogranin A**	**MIB1**
(A)	Round component	++	++	++	+	-	25%
(B)	Crescent component	+	-	-	-	-	50%

LCA, L26, UCHL1, CD3, ASMA, M-actin, desmin, CD34, NF, GFAP, and S100 were negative in both components.

The patient was discharged three weeks after operation with uneventful postoperative period. Four cycles of monthly adjuvant chemotherapy with 5-fluoro uracil (5-FU) (325 mg/m^2^) and leucovorin (20 mg/m^2^) were administered. The patient showed no sign of recurrence and is disease-free 48 months after surgery.

## Discussion

Neuroendocrine carcinomas (NEC) in the duodenum are extremely rare, and are classified as either 'small-cell' or 'non small-cell' types. The small-cell NEC occurring in the duodenum and elsewhere in gastrointestinal tract are similar to the small-cell carcinoma of the lung [7,8]. Only eight cases of small-cell NEC in the duodenum have been reported, with the present case being the ninth (Table 2) [7-12]. Most cases occurred in middle-aged or geriatric males with lesions in the ampulla of Vater. Extra-ampullary small-cell NECs in the duodenum are extremely rare, with only two cases reported previously [7,8].

**Table 2:** Profiles of the cases of primary small-cell neuroendocrine carcinoma in the duodenum reported in the literature.

	**Author Year**	**Age/sex**	**Clinical Manifestations**	**Size (mm)**	**Morphology**	**Location**	**Metastasis**	**Surgery**	**Chemotherapy**	**Prognosis (month)**
1	Swanson 1986 [7]	76 M	Abdominal pain, anorexia, weight loss	15	ulceration	adjacent to the ampulla*	LN, liver	biospy	5-FU, doxorubicin, mitomycin	Dead (1.5)
2	Zamboni 1990 [8]	62 M	jaundice, weight loss	25	polypoid	the papilla of Vater	LN	PD	-	Dead (7)
3	Zamboni 1990 [8]	66 M	jaundice, abdominal pain	20	ulceration	the papilla of Vater	LN	PD	-	Dead (6)
4	Zamboni 1990 [8]	51 M	jaundice, weight loss, abdominal pain	30	soft fungating mass	the papilla of Vater	LN	PD	-	Dead (17)
5	Lee 1992 [9]	86 M	jaundice, recurrent pancreatitis	?	polypoid	Peri ampullary	?	-	-	(>5)
6	Sarker 1992 [10]	53 F	jaundice, weight loss, back pain	35	mass with small ulceration	the papilla of Vater	LN	PD	5-FU, TNF, interferon	Recurrence + (>18)
7	Sato 1995 [11]	74 M	jaundice	35	polypoid	the papilla of Vater	?	PPPD	-	?
8	Shim 2000 [12]	54 M	jaundice	30	ulceration	the papilla of Vater	liver	PD	cisplatin, etoposide, radiation	Dead (8)
9	Sata 2004 present case	57 M	GI Tract bleeding	30	mass with ulceration	Peri ampullary	-	Local resection	5-FU leucovorin	disease free (>48)

* Hormone VIP; ? Don't know; M – male; F – Female; PD-pancreaticoduodenectomy; LN-lymph node; PPPD-pylorus preserving pancreaticoduodenectomy; 5 FU-f fluoro uracil; TNF – tumor necrosis factor

The natural course of small-cell NECs in the duodenum is still not clear. Most cases reported in the literature show rapid progress of the disease, with radical surgery and/or chemotherapy not altering the clinical course, and thus a poor prognosis. In six of the previous eight cases, patients underwent pancreaticoduodenectomy for removal of the tumor, while the remaining two did not undergo surgery because of multiple liver metastasis or poor general condition. In spite of radical resection with or without adjuvant chemotherapy, most cases showed rapid recurrence and metastasis. Of the eight reported cases, one was unusual as it occurred in a middle-aged female with rapid progress of the disease but effective response to adjuvant chemotherapy using 5FU, tumor necrosis factor, and interferon this patient survived for more than eighteen months [10]. The present case was treated by a local excision of the tumor followed by adjuvant chemotherapy using 5-FU and leucovorin, and showed a distinctively unique clinical course, with the patient surviving for more than 48 months without any sign of recurrence. This case was presented with gastrointestinal bleeding, which contributed to early diagnosis, whereas the other previous cases in literature presented either with abdominal pain or jaundice. Hence, the good prognosis in present case could also be due to its earlier presentation. The lesion in the present case showed a different immunohistological character from that in other cases, such as no immunoreactivity to neuron-specific enolase (NSE) or chromogranin A. These differences too might partly explain the different character of this case.

## Conclusions

This report identifies a new subtype of small-cell NEC in the duodenum. This subtype appears to have a much better prognosis, and may be amenable to local excision, if the lesion is away from the ampulla of Vater.

## Competing interests

None declared.

## Authors' contributions

NS, MT, MK, KY, KK, HN took part in the operation, performed the literature search and drafted the manuscript for submission. HN supervised the preparation of the manuscript and edited the final version for publication. TS, KS performed pathological investigations and contributed to the pathological content of the manuscript.

All authors read and approved the manuscript.
